# Olfactory neuroepithelium in the middle turbinate: is there any impact on olfaction function after lateral marsupialization for concha bullosa surgery?

**DOI:** 10.1016/j.bjorl.2021.06.005

**Published:** 2021-07-20

**Authors:** Onur İsmi, Feyzi Meşe, Harun Gür, İclal Gürses, Yusuf Vayısoğlu, Kemal Görür, Cengiz Özcan

**Affiliations:** a University of Mersin, Faculty of Medicine, Department of Otorhinolaryngology, Mersin, Turkey; b Special Batman Dünya Hospital, Otorhinolaryngology specialist, Batman, Turkey; c University of İstanbul, Cerrahpaşa Faculty of Medicine, Department of Pathology, İstanbul, Turkey

**Keywords:** Olfaction, Olfactory membrane protein, Concha bullosa, Middle turbinate concha bullosa, Lateral marsupialization

## Abstract

**Introduction:**

The effect of the quantity of olfactory neuroepithelium in the middle turbinate on the postoperative olfactory function for middle turbinate concha bullosa patients has not yet been evaluated.

**Objective:**

The primary aim of this study was to investigate the olfactory structures in the middle turbinate by immunohistochemical analysis of the olfactory marker protein and to correlate the immunostaining results with the olfaction test results for patients with middle turbinate concha bullosa.

**Methods:**

Surgical materials of 18 middle turbinate concha bullosa patients who had undergone lateral marsupialization surgery were immunostained with olfactory marker protein antibodies. Smell diskettes olfaction test was applied to all of the study group patients both preoperatively and three months postoperatively. A visual analog scale was used to quantify the sense of nasal obstruction.

**Results:**

It was observed that the postoperative smell scores and the nasal obstruction visual analog scale values were significantly improved as compared to the preoperative values (*p* < 0.05). In addition, there was a significant correlation between the smell score gain and the visual analog scale gain values (r = 0.682). Results also indicated no significant correlation between the olfactory marker protein staining scores and the smell scores (*p* > 0.05).

**Conclusion:**

This first paper demonstrated that the quantity of the olfactory mucosa in the middle turbinate was not a determining factor for the postoperative smell function degree for middle turbinate concha bullosa patients. The underlying cause of the olfactory deficit for middle turbinate concha bullosa patients seems to be obstruction related rather than the middle turbinate’s olfactory mucosa containing status.

## Highlights

• Lateral marsupialization is an effective method for restoration of both smell function and nasal breathing in concha bullosa patients.
• The presence and quantity of olfactory mucosa in the middle turbinate may not significantly affect the postoperative smell function.
• The underlying cause of olfactory deficit in middle turbinate concha bullosa patients seems to be middle meatus obstruction.

## Introduction

Concha bullosa (CB) is one of the most common anatomic variations of the sinonasal region, characterized by the presence of an air cell in the turbinate.^1^, ^2^ It is most commonly seen in the middle turbinate, whereas the superior and inferior turbinates are rarely involved.^3^ The underlying etiopathogenesis is not actually known. Expansion of the paranasal sinus pneumatization into the middle turbinate, fusion abnormality in the embryological period and bone microfractures in the turbinate at late puberty period are possible proposed mechanisms.^2^ According to the Bolger classification,^4^ the middle turbinate CB can be categorized as: (a) lamellar type when the pneumatization is limited to the vertical lamella, (b) bulbous type when the pneumatization includes the inferior bulbous portion and (c) extensive or true type when the pneumatization is present in the entire turbinate. Middle turbinate CB is regarded as a variation of the lateral nasal wall rather than a pathological condition. However, it has been shown that the extensive type CB can cause ostiomeatal complex obstruction and rhinosinusitis. Although most of the patients are asymptomatic and they are diagnosed incidentally during the radiological evaluation, a larger sized CB can cause undesired symptoms such as nasal obstruction, headache and pressure sensation.^1^, ^5^

Smell dysfunction is an underestimated symptom in patients with CB. The definitive treatment of the symptomatic CB is surgery, however, the primary aims of surgery include maintaining the patency of the nasal passage and eliminating the pressure sensation and obstruction of the ostiomeatal complex. A limited number of studies have focused on the olfactory function results after surgical management of CB patients via different operative techniques.^1^, ^5^, ^6^ Olfactory marker protein (OMP) is a neuropeptide found in the cell bodies and peripheral structures of the olfactory receptor neurons in the olfactory neuroepithelium. Antibodies against OMP were widely used to detect the olfactory structures in the nasal cavity for evaluation of rhinological disorders.^7^, ^8^ It has been reported that most middle turbinate biopsies contain olfactory neuroepithelium when the CB surgical materials are immunostained with the anti-OMP antibodies.^7^ To the best of authors’ knowledge, the impact of the quantity of the olfactory mucosa in the middle turbinate on the postoperative olfactory function for middle turbinate CB patients has not yet been explored or evaluated in depth.

In this study, we aimed to evaluate the smell function of patients presenting with the extensive type middle turbinate CB and undergoing endoscopic lateral marsupialization of the CB. The smell diskettes olfaction test was used for the objective assessment. The olfactory structures in the middle turbinate of the patients were analyzed by the immunohistochemical staining of OMP in the surgical materials. Accordingly, the correlation analysis was employed between the staining quantities and the preoperative/postoperative olfaction test results. Furthermore, the degrees of preoperative and the postoperative nasal obstruction of the patients were assessed with the visual analogue scale (VAS) and a systematic correlation analysis was carried out between the VAS score gain values and the smell score gain values.

## Methods

Local ethical committee approval was acquired for the current study (ethical committee approval nº 2010351). The study group consisted of extensive type middle turbinate CB patients who had undergone endoscopic lateral marsupialization surgery in our tertiary center otorhinolaryngology clinic. The diagnosis of CB was made according to the physical examination and maxillofacial coronal computerized tomography (CT) findings. The primary complaint of all of the study group patients was unilateral nasal obstruction for more than three months and this symptom was the main indication for the lateral marsupialization surgery. All patients underwent a detailed clinical examination including anterior rhinoscopy, nasal endoscopy with rigid telescopes (0 or 30°) (Karl Storz®, Germany) and paranasal sinus CT. Atopy status of the patients were evaluated using the skin prick test with a standardized allergen prick test panel (Stallergenes®, Antony, France).^9^ Only skin prick test negative patients were taken to the study to eliminate the effect of atopy on the study results. The exclusion criteria also included: pediatric patients (<18 years of age), nasal septum deviation, nasal polyposis, previous nasal surgeries and mental and neurological disorders.

### Lateral marsupialization surgical technique

All of the patients included in this study were operated under general anesthesia via the endoscopic approach by the same surgical team. The 4 mm 0° endoscope (Karl Storz®, Germany) was used during the lateral marsupialization surgery of all of the study group patients. After topical anesthetic (20 mg/mL Lidocaine and 0.0125 mg/mL epinephrine, Jetokain®, Adeka Drugs, Samsun, Turkey) injection, entrance to the middle turbinate was accomplished from the most prominent part with the help of a sickle knife. The incision was continued with the concha scissor or the sickle knife through the free lower edge of the middle turbinate. Through-cutting forceps were used to cut the lateral part of the middle concha and to gently excise it. Merocel® nasal tampons were placed between the lateral nasal wall and the remnant middle turbinate in order to prevent the lateralization of the turbinate, synechia formation and postoperative bleeding. The nasal packs were removed on the second postoperative day. Postoperative complications such as bleeding, synechia formation or cerebrospinal fluid (CSF) leakage were not seen in any of the study group patients. None of the patients underwent any additional surgical procedure including septoplasty or inferior turbinate radiofrequency during lateral marsupialization of the CB. A standardized postoperative medical treatment protocol including amoxicillin–clavulanate 1 g twice a day, paracetamol 500 mg three times a day, cetirizine 10 mg once a day by oral route was applied to all of the patients after surgery until the removal of the nasal packs. Nasal irrigation with saline solution was employed after the removal of the nasal packs for two weeks.

The key parameters that were used for evaluation and comparison purposes in this study were as follows:

Nasal obstruction scores (visual analogue scale): due to the fact that the results of the VAS correlate with the rhinomanometry results for patients having nasal obstruction,^10^ CB patients were asked to quantify their sense of nasal obstruction based on a VAS.^5^, ^10^ The degree of nasal obstruction ranged from score 0 (no obstruction) to score 10 (complete obstruction). The VAS values were evaluated preoperatively and three months after the surgery. In addition, the nasal obstruction (VAS) gain results were measured by subtracting the postoperative VAS values from the preoperative ones.

Smell scores: the smell diskettes olfaction test (Novimed, Dietikon, Switzerland) was used as the standardized and validated smell identification screening test. As previously described, eight odorants were used in a high suprathreshold concentration for the olfaction test. The patient received a score between 0 (no smelling) and 8 (optimal smelling) based on the olfaction performance.^11^, ^12^ The smell test was performed in the preoperative period and three months after the lateral marsupialization surgery for each patient. Similarly, smell score gain values were measured by subtracting the preoperative smell scores from the postoperative ones.

Immunohistochemistry (Immunostaining with the OMP antibody): middle turbinates’ surgical excision materials were fixed in 10% formaldehyde, embedded in paraffin, and cut into 4 µm sections. One section was systematically stained for routine hematoxylin–eosin pathological examination. Sections which were processed for immunohistochemistry were deparaffinized and blocked with 4% bovine serum phosphate-buffered saline (PBS) for immunohistochemical analysis. Sections were incubated with antibodies against olfactory marker protein (OMP Antibody, Abcam, ab62144). After 20 min of pretreatment, the sections were cooled at room temperature for 20 min and washed with distilled water. After another rinse with the PBS solution, the peroxidase block solution was applied for 10 min. Subsequently, the protein block solution and another rinse with PBS solution were employed and the primary antibodies against OMP were exposed. Afterwards, another rinse with PBS solution was applied, and the sections were treated with the diaminobenzidine solution. Finally, counterstaining with hematoxylin was performed and sections were dehydrated in 96% alcohol and covered with Ultramount Labvision balsam for histopathological analysis.^7^

**Figure 1:**
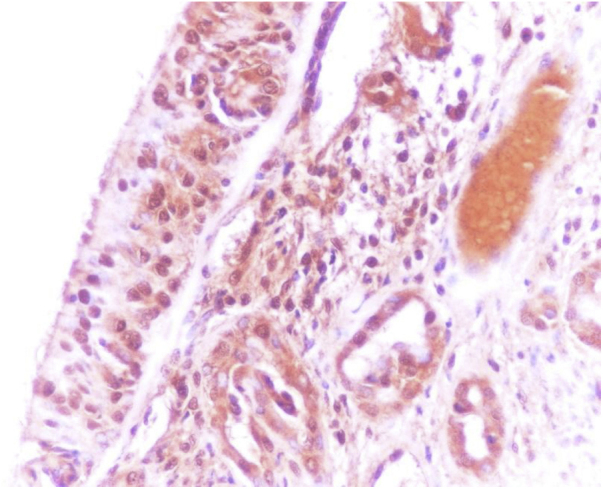
Very severe (+4) immunostaining of olfactory epithelium with the olfactory marker protein (OMP, ×400).

**Figure 2:**
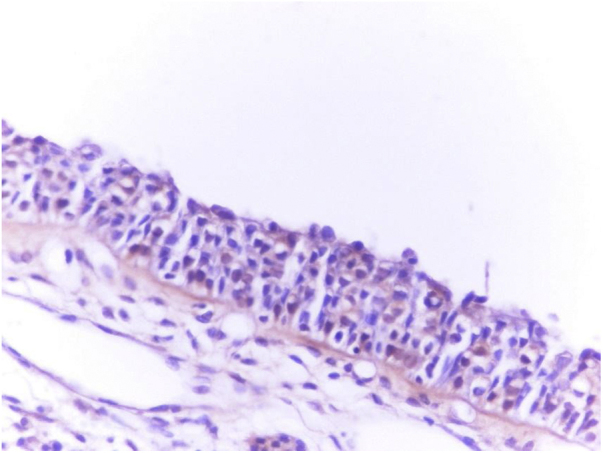
Severe (+3) immunostaining of concha bullosa surgical material with the olfactory marker protein (OMP, ×400).

All pathological specimens were evaluated by the same pathologist. For each specimen, the quantity of the immunostaining was analyzed. Staining quantity was scored from score 0 (no staining) to 4 (very severe staining) for each patient.^13^ The olfactory epithelium was regarded as the positive control (score 4 staining) for OMP immunostaining. The olfactory epithelium was obtained from another patient with nasal polyps who underwent endoscopic sinus surgery (ESS) from the dorsoposterior part of the nasal cavity at the upper part of the superior turbinate^14^ (Figure 1, Figure 2).

### Statistical analysis

Statistical analysis was performed using SPSS version 24.0 (IBM SPSS, New York, USA, 2016). Data were shown in terms of mean ± standard deviation for continuous variables and the number of cases were used for categorical ones. Data were controlled for normal distribution using the Shapiro–Wilk test. The paired sample *t*-test was used to compare the mean preoperative/postoperative smell scores and the nasal obstruction VAS values of the patients. The Pearson correlation coefficient was employed to evaluate the correlation between the mean smell score gain values and the nasal obstruction VAS gain values. In addition, the Pearson correlation coefficient was also used to analyze the correlation between the mean OMP staining scores and the pre/postoperative smell scores; *p*-value of <0.05 was regarded as statistically significant.

## Results

There were 18 patients in the study group. Among these patients, 11 (61.1%) were male and 7 (38.9%) were female. The mean of the age of the study group patients was 29.8 ± 11.95 years (min = 18, maximum = 62).

**Figure 3:**
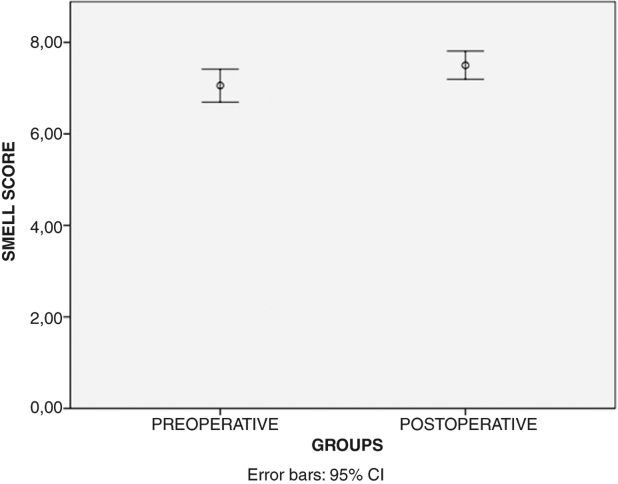
Comparison of the mean smell test scores after application of the smell diskettes olfaction test was illustrated.

The means of the preoperative and postoperative smell scores were found to be 7.05 ± 0.72 and 7.5 ± 0.61, respectively. It was observed that postoperative smell scores were significantly higher as compared to the preoperative smell scores (*p* = 0.007) (Fig. 3).

**Figure 4:**
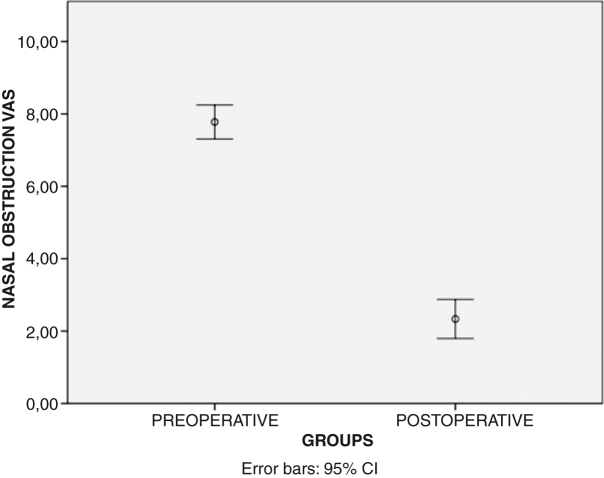
Comparison of the mean nasal obstruction visual analogue scale values before and after the surgery was presented. (VAS, visual analogue scale).

Similarly, the means of the preoperative and the postoperative nasal obstruction VAS values were found to be 7.77 ± 0.94 and 2.33 ± 1.08, respectively. The results indicated that postoperative VAS values were significantly lower as compared to the preoperative values (*p* < 0.001) (Fig. 4).

**Figure 5:**
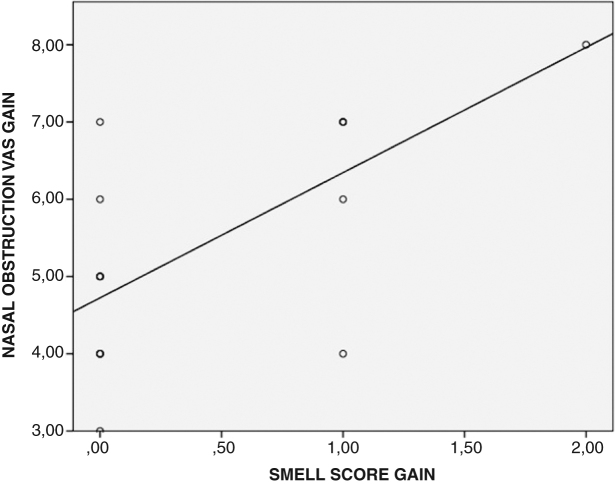
Correlation between the smell score gain and the visual analogue scale gain values of the patients was demonstrated. (VAS, visual analogue scale).

The Pearson correlation analysis revealed that there was a significant correlation between the smell score gain values and the nasal obstruction VAS gain values (r = 0.682, *p* = 0.002) (Fig. 5).

Fifteen (83.3%) patients exhibited positive staining with OMP antibodies in the surgical materials of the middle turbinate. One (5.5%) patient had mild (score 1), 6 (33.3%) patients had moderate (score 2) and 8 (44.4%) patients had shown severe (score 3) staining based on the staining quantities with the OMP antibodies. There was no patient with very severe (score 4) staining. The mean of the staining scores of the patients was found to be 2.05 ± 1.1 (Table 1).

**Table 1:** Olfactory marker protein staining scores of the patients.

Staining score	Number of patients (n/%)
0 (No staining)	3/16.6%
1 (Mild staining)	1/5.5%
2 (Moderate staining)	6/33.3%
3 (Severe staining)	8/44.4%
4 (Very severe staining)	0/0%
Mean: 2.05 ± 1.1	

The Pearson correlation analysis revealed that there was no significant correlation between the OMP staining scores and the preoperative smell scores (r = −0.369, *p* = 0.131). Similarly, the Pearson correlation analysis also exhibited that there was no significant correlation between the OMP staining scores and the postoperative smell scores (r = −0.043, *p* = 0.866).

## Discussion

In this study, we found that the lateral marsupialization technique appeared to restore the smell function and the sense of nasal breathing in extensive type middle turbinate CB patients. Moreover, the smell score gain values were significantly correlated with the nasal obstruction VAS gain values. It was also observed that most of the lateral marsupialization surgical materials contained olfactory neuroepithelium of moderate-severe degrees. However, partial surgical excision of these turbinates did not result in impairment of the olfaction function and even improved the smell scores after surgery.

There is still an ongoing debate on the exact arrangement of the olfactory neuroepithelium in the nasal cavity. In the 1980s, the olfactory epithelium was believed to be restricted to the olfactory cleft area located at the superior part of the nasal cavity between the nasal septum, the cribriform plate and the superior turbinate.^15^, ^16^ However, during the early periods of the 21st century, it was demonstrated that the olfactory neuroepithelium could be localized even at 22 mm. anterior to the olfactory cleft area.^17^ Recently, Apuhan et al. presented that approximately 82% of the middle turbinate biopsies of CB patients contained olfactory mucosa. In particular, the lateral part of the turbinate was found to contain olfactory epithelium more frequently, compared to the anterior and the medial parts.^7^ Our study results were consistent with the results of the study of Apuhan et al., with an 83.3% rate of OMP-positive staining CB surgical materials. Hence, according to our results and the reported data in the literature, it is reasonable to state that the olfactory neuroepithelium is not restricted to the olfactory cleft area. It can also be found in the anterior parts of the nasal cavity including the middle turbinate in most patients with CB.

Middle turbinate CB is mostly asymptomatic, however, a large CB can cause nasal obstruction, headache, rhinosinusitis, and olfactory deficit. The frequency of CB has been reported to be 5.7%–55% in symptomatic cases and 10%–20% in asymptomatic cases.^1^ There are four different surgical techniques defined for the surgical management of symptomatic middle turbinate CB, namely, lateral marsupialization, medial marsupialization, transverse resection and crushing methods.^5^ The lateral marsupialization technique in which the lateral part of the middle turbinate is partially resected is the most common applied surgical method. This technique can eliminate the nasal obstruction and ostiomeatal complex blockage, however formation of synechia between the middle turbinate remnant and the lateral nasal wall can be observed as a complication.^1^, ^5^ The lateral marsupialization technique was performed on all of the patients without any complications in our study. According to our results, the lateral marsupialization technique was found to be an effective and safe method for middle turbinate CB patients. It was observed that, this technique not only increased the sense of nasal breathing but also increased the olfaction function after CB surgery.

Various studies have focused on the effects of the middle turbinate surgeries on the olfactory function. Middle turbinate medialization and controlled synechia formation with the nasal septum has been used to prevent ostiomeatal complex obstruction and middle turbinate lateralization during ESS.^18^, ^19^ Theoretically, this technique can prevent the airflow to the olfactory cleft area and diminish the smell function after surgery. However, it was observed that the middle turbinate medialization technique had no adverse effect on the olfaction scores after ESS.^19^, ^20^ Resection of the middle turbinate is another surgical technique for preventing lateralization of the turbinate during ESS. Some researchers argue that resection of the middle turbinate decreases the destabilization of the turbinate. Furthermore, they claim that resection increases the airflow into the ethmoid labyrinthine and prevents the ostiomeatal complex obstruction and synechia formation. On the other hand, resection of the turbinate may lead to frontal rhinosinusitis, atrophic rhinitis and empty nose syndrome.^21^ With regard to the olfactory outcomes, compared to the preservation technique, the resection of the middle turbinate does not decrease the olfactory scores and even improves them after ESS.^22^, ^23^

A small number of studies have investigated the olfaction function outcomes after middle turbinate CB surgeries. Kilicaslan et al.^6^ reported increased smell scores after middle turbinate resection and reduction surgeries combined with septoplasty in patients with nasal septum deviation and CB. Recently, Akkoca et al.^1^ compared the crushing and lateral marsupialization techniques in patients with middle turbinate CB. They reported that both techniques increased the postoperative olfactory scores; however, the crushing technique increased the smell scores more significantly compared to the lateral marsupialization method. Similarly, Andaloro et al.^5^ found that lateral marsupialization and crushing techniques were both effective for restoring the olfactory deficit in patients with middle turbinate CB, however on the contrary they found no significant difference in the olfactory gain values between these two techniques.^1^ None of these studies investigated the presence of olfactory structures in the middle turbinate and the effect of these structures on the smell scores after surgery in middle turbinate CB patients. According to our study results, the vast majority of turbinate specimens of patients with CB were found to contain olfactory structures, however partial resection of these conchae did not reduce the smell function and even improved it. No correlation was found between the quantity of the olfactory neuroepithelium in the middle turbinate and the pre/postoperative smell scores. Furthermore, our study results put forth the presence of a significant correlation between the smell score gain values and the nasal obstruction VAS gain values. These findings indicate that the underlying cause of olfactory deficit in patients with CB seems to be the airflow blocking effect of the CB rather than the quantity of the olfactory neuroepithelium in the middle turbinate. As widely known, the internal nasal valve is the region with the least cross-sectional area in the whole nasal airway and minor obstruction in this region can result in increased resistance and decreased nasal flow. However, a recent computerized model study demonstrated that most of the nasal airflow passed from the middle meatus rather than the inferior meatus after passing through the internal nasal valve region.^24^ Hence, a large middle turbinate CB can block the middle meatus and prevent the nasal airflow resulting in symptomatic CB with nasal obstruction. Secondly, a large middle turbinate CB can also prevent the turbulence airflow towards the olfactory cleft region. Recently, a computerized fluid dynamics study of the nasal airway^25^ demonstrated an increased influx of air into the olfactory cleft area after middle turbinate resection. Thus, partial resection of the turbinate may restore the turbulence airflow towards the olfactory cleft area and may overcome the olfactory deficit despite the presence of the olfactory neuroepithelium in the middle turbinate in CB patients.

A limitation of our study was the small number of patients. To evaluate the effect of surgical excision of the olfactory epithelium containing CB specimens on the olfactory function, we could only include patients who had undergone lateral marsupialization surgery without septoplasty or inferior turbinate radiofrequency. However, we think that our results are meaningful with regard to the relationship between the olfactory epithelium containing status of the middle turbinate and the postoperative smell function in patients with symptomatic CB. Future studies with larger numbers of participants may accurately determine the minimal clinically important difference value for Smell Diskettes Olfaction Test which would be used for objective assessment of the olfactory function in patients with symptomatic CB.

## Conclusion

Our study suggested that lateral marsupialization technique is an effective method, not only for the nasal breathing sensation but also for restoration of smell function in symptomatic middle turbinate CB patients. Most (83.3%) of the CB samples were found to contain olfactory epithelium after immunostaining with OMP antibodies in our study, however there was no correlation between the immunostaining scores and the smell scores based on the Smell Diskettes Olfaction test results. These findings suggest that the presence and the quantity of olfactory mucosa in the middle turbinate may not significantly affect the postoperative smell function in middle turbinate CB patients. The underlying cause of olfactory deficit in middle turbinate CB patients seems to be middle meatus obstruction, since the nasal obstruction score gain values were directly correlated with the smell score gain values in our study.

## Informed consent

Written informed consent was taken from all patients who participated in the study.

## Ethical committee approval

Local ethical committee approval was acquired for the current study.

## Funding

This study was funded by Academic research unit committee of 10.13039/501100004172Mersin University.

## Conflicts of interest

The authors declare no conflicts of interest.
